# Deep Red Photoluminescence from Cr^3+^ in Fluorine-Doped Lithium Aluminate Host Material

**DOI:** 10.3390/ma17020338

**Published:** 2024-01-10

**Authors:** Yuki Kamada, Ryusei Hayasaka, Kento Uchida, Taisei Suzuki, Takahiro Takei, Mamoru Kitaura, Hiroko Kominami, Kazuhiko Hara, Yuta Matsushima

**Affiliations:** 1 Applied Chemistry, Chemical Engineering, and Biochemical Engineering, Yamagata University, Yonezawa 992-8510, Japan; 2 Center for Crystal Science and Technology, University of Yamanashi, Kofu 400-0021, Japan; takei@yamanashi.ac.jp; 3 Faculty of Science, Yamagata University, Yamagata 990-8560, Japan; kitaura@sci.kj.yamagata-u.ac.jp; 4 Faculty of Engineering, Shizuoka University, Hamamatsu 432-8561, Japan; kominami.hiroko@shizuoka.ac.jp; 5 Research Institute of Electronics, Shizuoka University, Hamamatsu 432-8011, Japan; hara.kazuhiko@shizuoka.ac.jp

**Keywords:** deep red phosphor, fluorine-doped lithium aluminate, spinel compound, disordered spinel, 3d transition metal ion

## Abstract

Deep red phosphors have attracted much attention for their applications in lighting, medical diagnosis, health monitoring, agriculture, etc. A new phosphor host material based on fluorine-doped lithium aluminate (ALFO) was proposed and deep red emission from Cr^3+^ in this host material was demonstrated. Cr^3+^ in ALFO was excited by blue (~410 nm) and green (~570 nm) rays and covered the deep red to near-infrared region from 650 nm to 900 nm with peaks around 700 nm. ALFO was a fluorine-doped form of the spinel-type compound LiAl_5_O_8_ with slightly Li-richer compositions. The composition depended on the preparation conditions, and the contents of Li and F tended to decrease with preparation temperature, such as Al_4.69_Li_1.31_F_0.28_O_7.55_ at 1100 °C, Al_4.73_Li_1.27_F_0.17_O_7.65_ at 1200 °C, and Al_4.83_Li_1.17_F_0.10_O_7.78_ at 1300 °C. The Rietveld analysis revealed that ALFO and LiAl_5_O_8_ were isostructural with respect to the spinel-type lattice and in a disorder–order relationship in the arrangement of Li^+^ and Al^3+^. The emission peak of Cr^3+^ in LiAl_5_O_8_ resided at 716 nm, while Cr^3+^ in ALFO showed a rather broad doublet peak with the tops at 708 nm and 716 nm when prepared at 1200 °C. The broad emission peak indicated that the local environment around Cr^3+^ in ALFO was distorted, which was also supported by electron spin resonance spectra, suggesting that the local environment around Cr^3+^ in ALFO was more inhomogeneous than expected from the diffraction-based structural analysis. It was demonstrated that even a small amount of dopant (in this case fluorine) could affect the local environment around luminescent centers, and thus the luminescence properties.

## 1. Introduction

Inorganic phosphors have been widely used in display and lighting applications such as cathode-ray tubes and fluorescent lamps. Conventional phosphors used in these applications are typically excited by high-energy radiation such as X-rays and ultraviolet (UV) rays or electron beams. Recent light-emitting diode (LED) lighting achieves a pseudo-white color by combining blue light emitted from LED chips and the emission of the phosphors excited by the blue light, which has lower energy than X-rays and UV rays.

“Red” is an important color in lighting applications because it has a significant emotional effect on humans, as people unconsciously perceive the warmth of an atmosphere, the state of a person’s health from their complexion, or the freshness of red meat from the color red. Typical rare-earth ions that emit red light are Eu^3+^ and Eu^2+^. The former shows orange-red luminescence with a sharp peak around 610 nm due to the 4f-4f transition in some host materials, while the latter, e.g., CaAlSiN_3_:Eu^2+^ [1,2,3] and K_2_CaPO_4_F:Eu^2+^ [4], shows a rather broad emission between 600 and 700 nm due to the 5d-4f transition. In recent years, fluorescent materials other than inorganic phosphors, such as polymethine dye molecules, their aggregates and nanoparticles, conjugated polymer nanoparticles, single-walled carbon nanotubes, quantum dots, and lanthanide complex molecules, have also been extensively investigated for emerging applications utilizing near-infrared emission from 700 to 2000 nm, including medical diagnosis, health monitoring, agriculture, and night vision [5,6,7,8].

The 3d transition metal ions, Cr^3+^, Mn^4+^, and Fe^3+^, can act as luminescent centers emitting in the red to deep red region from 640 to 800 nm in suitable host materials due to 3d-3d transitions [9]. The luminescence properties are highly dependent on the combination of the luminescent center ions and the host materials. For example, Mn^4+^ in K_2_SiF_6_ has sharp excitation lines around 450 nm, leading it to be a candidate for a red phosphor for LED lighting [10]. On the other hand, Mn^4+^ in SrLaAlO_4_ [11] and SrGdAlO_4_ [12] is not excited by blue light as it shows the excitation peak in the UV region around 365 nm.

Cr^3+^ has specifically attracted attention as a luminescent center for near-infrared luminescence. Various compounds, whether natural or synthetic, have been reported as host materials, in which Cr^3+^ often replaces Al^3+^ or Ga^3+^ in the host compounds. The sharp deep red emission around 694 nm of Cr^3+^ in α-Al_2_O_3_ has been utilized for solid-state lasers. Emission of Cr^3+^ has also been observed in, for example, β-Ga_2_O_3_ [13], spinel-type MgAl_2_O_4_ [14,15,16], ZnAl_2_O_4_ [15,16,17,18], LiAl_5_O_8_ [19,20], garnet-type Y_3_Al_5_O_12_ [21,22,23], Gd_3_Ga_5_O_12_ [24], and Gd_3_Sc_2_Ga_3_O_12_ [22], beryl Be_3_Al_2_Si_6_O_18_ [25,26], aluminosilicate CaSc_1–x_Al_1+x_SiO_6_ [27], SrAl_11.88−*x*_Ga*_x_*O_19_ [28], Bi_2_Ga_4_O_9_ [29], La_3_GaGe_5_O_16_ [30], and phosphate K*M*P_2_O_7_ (*M* = Ga, Sc, In, and Lu) [31]. Cr^3+^ in these host materials has been considered to occupy octahedral sites. When the strength of the crystal filed is relatively large, such as α-Al_2_O_3_ and the spinel-type hosts [14,15,16,17,18,19,20], sharp emission spectra are observed near 700 nm due to the electronic transition from the excited state ^2^*E* to the ground state ^4^*A*_2_, while in the host materials with a weak crystal field, such as the garnet-type hosts [21,22,23,24], CaSc_1−x_Al_1+x_SiO_6_ [27], Bi_2_Ga_4_O_9_ [29], and La_3_GaGe_5_O_16_ [30], a red-shifted broad emission is observed due to the spin-allowed ^4^*T*_2_ → ^4^*A*_2_ transition.

We have demonstrated different types of deep red luminescence from 650 to 800 nm achieved with a single host material doped with either Mn^4+^, Fe^3+^, or Cr^3+^ [32]. The host material was prepared from LiF and Al_2_O_3_ at 1:2, which first appeared in the report by Belov et al. [33], described as “Al_4_LiO_6_F” with a spinel-type lattice, assuming a stoichiometric reaction between LiF and two Al_2_O_3_. Our previous paper and other researchers referred to this material as Al_4_LiO_6_F [34] or LiAl_4_O_6_F [35], following Belov’s suggestion. However, the composition of Al_4_LiO_6_F does not match the spinel-type composition of *AB*_2_O_4_. In normal spinel, the divalent cation *A*^2+^ and the trivalent cation *B*^3+^ occupy the tetrahedral and octahedral cation sites in the spinel-type lattice.

In our previous paper [32], we showed that Mn^4+^ in the host material prepared from LiF and Al_2_O_3_ significantly enhanced photoluminescence (PL) compared to spinel-type lithium aluminate LiAl_5_O_8_, indicating its potential as a new host material for deep red phosphors. However, the details of the composition and crystal structure had not been clarified. In this paper, we denote this host material as aluminum lithium fluoride oxide (ALFO) and discuss the characteristics of ALFO and the luminescent properties of Cr^3+^ in ALFO, the luminescent center ion for deep red phosphors which can be excited by visible radiation; this finding will help provide new insights into the design of new phosphor host materials.

## 2. Materials and Methods

ALFO samples were prepared from LiF and γ-Al_2_O_3_; LiF was prepared from lithium hydroxide monohydrate (99%, Kanto Chem. Co., Inc., Tokyo, Japan) and ammonium fluoride (99%, Kanto Chem. Co., Inc.) by precipitation in aqueous solution. The starting aluminum source was aluminum triisopropoxide (>99.9%, Kanto Chem. Co., Inc.); aluminum hydroxide was precipitated at pH 10 in isopropanol solution by NH_3_ aq. Using Cr(NO_3_)_3_·9H_2_O (98.0–103.0%, Kanto Chem. Co., Inc.) as the starting material, Cr^3+^ was coprecipitated with aluminum hydroxide in the isopropanol solution. The coprecipitated hydroxide mixture was rinsed with deionized water, heated in a platinum crucible to 1000 °C at a ramp rate of 10 °C/min, and cooled in the furnace to obtain γ-Al_2_O_3_:Cr^3+^. The concentration of Cr^3+^ was defined as Cr^3+^/(Al^3+^ + Cr^3+^), assuming that it replaced Al^3+^ in the products. γ-Al_2_O_3_:Cr^3+^ and LiF were weighed at (Al + Cr)/Li = 4 (note that γ-Al_2_O_3_ contains a certain amount of water in its composition and that (Al + Cr)/Li in the starting composition could be slightly smaller than 4). Typically, 0.2 g of a mixture of LiF and γ-Al_2_O_3_:Cr^3+^ was placed in a 5 cm^3^ platinum crucible with a lid. The 5 cm^3^ platinum crucible was put in a larger platinum crucible (30 cm^3^) with a lid to suppress volatilization of the components, specifically lithium and fluorine, during heat treatment. Samples were heated at 800–1300 °C at a ramp rate of 20 °C/min. Heating times were 15 min at 1100 °C and above, and 1 h at 800–1000 °C.

For comparison, LiAl_5_O_8_:Cr^3+^ was prepared from Li_2_CO_3_ (99%, Kanto Chem. Co., Inc.) and α-Al_2_O_3_:Cr^3+^ at a ratio of Li:(Al + Cr) = 1:5. α-Al_2_O_3_ was prepared by heating the Al(OH)_3_ precipitate at 1200 °C for 2 h in air. The Li and Al sources were thoroughly mixed in an alumina mortar and pressed into pellets. The pellets were heated in an alumina crucible with the inner bottom covered with pre-prepared LiAl_5_O_8_ powder to suppress the diffusion of the lithium component from the sample to the alumina crucible.

The chemical compositions of the samples were analyzed for Li, Al, and Cr using inductively coupled plasma-optical emission spectroscopy after the samples were decomposed in molten salt. Fluorine contents were analyzed by ion chromatography after thermal hydrolysis. Oxygen contents were estimated from charge neutrality using the analyzed Al^3+^, Li^+^, Cr^3+^, and F^−^ contents. The morphology of the phosphor particles was observed with scanning electron microscopy (SEM) (T-330, JEOL Ltd., Tokyo, Japan and TM3030 plus, Hitachi High-Tech Co., Tokyo, Japan). Carbon was evaporated for conductive coating on the samples.

The crystallographic phases were identified by X-ray diffraction (XRD) using Cu Kα radiation (MiniFlex, Rigaku Co., Tokyo, Japan) operated at 40 kV and 15 mA. The scan step was 0.01°. Structural refinement was carried out by the Rietveld method using Rietan-FP [36]. The crystal structures were illustrated using VESTA [37]. The PL spectra and the photoluminescence excitation (PLE) spectra were measured using a fluorescence spectrometer (FL-6000, Shimadzu Co., Kyoto, Japan) and a multichannel spectral analyzer (PMA-11, Hamamatsu Photonics Co., Hamamatsu, Japan) combined with a lamp and a bandpass filter (MC-570, Asahi Spectra Co. Ltd., Tokyo, Japan) as the excitation light source. Quantum efficiencies (QEs) were evaluated with FL-6000 using an integral sphere. X-band (9.13 GHz) electron spin resonance (ESR) measurements were conducted at room temperature between 0 and 800 mT with 100 kHz magnetic field modulation to detect differences in the local environments around Cr^3+^ in the different host materials. As an aid for the discussion of the ESR results, classical molecular dynamics (MD) simulations using the MXDORTO code [38] were performed to reproduce the local structures. One Cr^3+^ ion was introduced into an octahedral site in the MD cell to replace the Al^3+^ ion, and the MD cell consisted of the crystallographic unit cells with *x*, *y*, and *z* axes of 23–26 Å. The detailed calculation procedure of the MD has already been described in our previous paper [39].

## 3. Results and Discussion

### 3.1. Compositions and Crystal Structure

**Figure 1:**
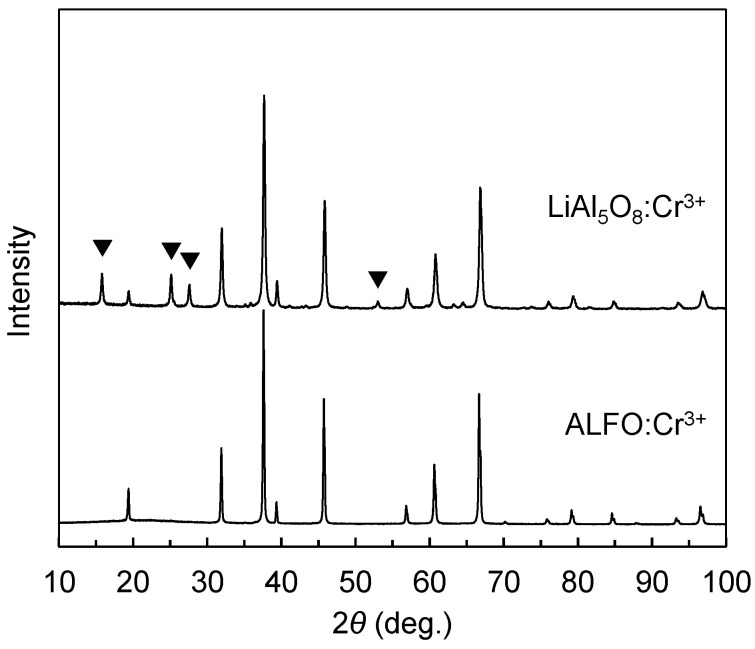
Comparison of XRD patterns of ALFO:Cr^3+^ and LiAl_5_O_8_:Cr^3+^ prepared at 1200 °C. The peaks marked with ▼ are the extra peaks originating in the cation ordering of Li^+^ and Al^3+^ in the spinel-type lattice of LiAl_5_O_8_ (see text for details).

Figure 1 compares the XRD patterns of ALFO:Cr^3+^ and LiAl_5_O_8_:Cr^3+^ prepared at 1200 °C with the Cr^3+^ concentration of 0.5%. Each pattern is consistent with the single phase of LiAl_5_O_8_ (ICDD # 38-1426) and Al_4_LiO_6_F (ICDD #38-610) reported by Belov et al. [33]. The peaks marked with ▼ are the extra peaks originating from the cation ordering of Li^+^ and Al^3+^ in spinel-type LiAl_5_O_8_, which will be discussed below.

Table 1 shows the chemical compositions of the ALFO host material without the luminescent center ion Cr^3+^ prepared at 1100 °C, 1200 °C, and 1300 °C. The XRD patterns of these samples are shown in Figure S1 in Supplementary Materials, confirming that each sample was in the single phase. The composition suggested by Belov et al. [33] was Al_4_LiO_6_F, but the compositional analysis (Table 1) showed that ALFO is a fluorine-doped form of LiAl_5_O_8_ with Li-rich compositions. The Al/Li/F ratios varied depending on the preparation temperature. The fluorine content was 0.28 for the sample prepared at 1100 °C; it decreased to 0.10 for the sample prepared at 1300 °C, indicating that fluorine dissipated during heat treatment at the high temperatures. The lithium content also decreased from Al/Li = 4.69/1.31 (=3.58/1) at 1100 °C and 4.73/1.27 (=3.72/1) at 1200 °C to 4.83/1.17 (=4.13/1) at 1300 °C.

The Cr^3+^ concentration in the Cr^3+^-doped ALFO samples was consistent with the starting composition; the compositional analysis showed that ALFO:Cr^3+^ prepared at 1200 °C with a starting concentration of 0.5% Cr^3+^ was 0.48%.

The structural refinement by the Rietveld analysis for ALFO prepared at 1200 °C without Cr^3+^ converged to *R*_wp_/*R*_p_ = 0.058/0.042, goodness-of-fit (*S*) = 1.90, and the lattice constant *a* = 7.9222(8) Å. Figure S2 in Supplementary Materials shows the refinement results and the structural parameters are listed in Table S1 in Supplementary Materials. The structural model was based on disordered spinel with the space group *Fd*−*3m*. The spinel-type lattice accommodates cations in the tetrahedral and octahedral sites. In the initial model, Al^3+^ and Li^+^ were assumed to be randomly distributed in the tetrahedral sites (8*a* position) and the octahedral sites (16*d* position). For the random distribution, the occupancies (*g*) of Li estimated from the chemical composition analysis (Table 1) should be *g* = 0.211 for both the tetrahedral and octahedral sites. However, the refined structure indicated a slight preference for Li to occupy the octahedral site, with *g* = 0.183 and 0.226 for the tetrahedral and octahedral sites, respectively.

**Figure 2:**
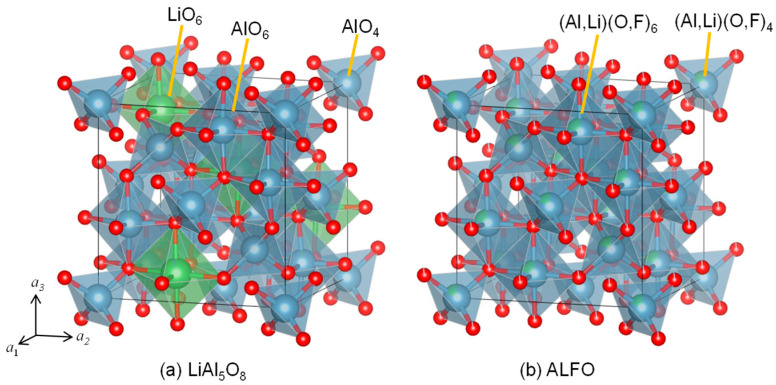
Crystal structures of (**a**) ordered LiAl_5_O_8_ and (**b**) aluminum lithium fluoride oxide (ALFO) after Rietveld analysis.

According to the literature, LiAl_5_O_8_ has crystallographic polymorphs, namely I (disordered) and II (ordered) [19,40,41,42] (Appendix A). Figure 2 compares the crystal structures of ALFO and ordered LiAl_5_O_8_. Figure S3 in Supplementary Materials illustrates the structural differences in the arrangement of the tetrahedra and the octahedra in the range of *x* = 0.2 to 0.6 for spinel-type ALFO and ordered LiAl_5_O_8_ in a perspective view along <100>. The octahedra form diagonal chains by edge sharing, and the chains of the octahedra are linked to each other by the tetrahedra connected by corner sharing. In ordered LiAl_5_O_8_, the tetrahedra consist of only AlO_4_, and the chains of the octahedra consist of one LiO_6_ and three AlO_6_ in sequence (Figure 2a and Figure S3a in Supplementary Materials). In ALFO, on the other hand, the tetrahedra are statistically AlO_4_ or LiO_4_ and the octahedra are AlO_6_ or LiO_6_ (Figure 2b and Figure S3b in Supplementary Materials).

The spinel-type framework leads to the unmarked peaks common to ALFO and LiAl_5_O_8_ in the XRD patterns in Figure 1. The peaks marked with ▼ in Figure 1 are derived from the periodicity due to the regular arrangement of Al^3+^ and Li^+^ on the cation sites in ordered LiAl_5_O_8_. The difference in the cation arrangement results in the lattice symmetry of *Fd*−*3m* for ALFO and *P4_3_32* for ordered LiAl_5_O_8_. The doped Cr^3+^ was assumed to occupy octahedral sites in both the host materials because of the preference of Cr^3+^ with the d^3^ configuration to occupy octahedral sites; the ionic radius of Cr^3+^ on a tetrahedral site is not defined in Shannon’s ionic radii table [43].

As a summary of the compositional and structural analyses, ALFO and ordered LiAl_5_O_8_ have a disorder–order relationship with respect to the arrangement of Li^+^ and Al^3+^ on the cation sites. ALFO has slightly Li-richer compositions than LiAl_5_O_8_, resulting in anion vacancies for the charge neutrality. Fluorine replaces 1.3–3.5% of the oxygen sites.

The solubility limit of Cr^3+^ in the ALFO lattice exceeded 10%. Figure S4 in Supplementary Materials shows the XRD patterns of ALFO:Cr^3+^ prepared at 1200 °C at different Cr^3+^ concentrations from 0.01 to 10%. All the samples were substantially a single phase of ALFO; the six-fold coordinated ionic radii of Al^3+^ and Cr^3+^ were 0.535 Å and 0.615 Å [43], and replacing Al^3+^ with larger Cr^3+^ shifted the diffraction peaks slightly toward the lower angles.

### 3.2. Morphologies

**Figure 3:**
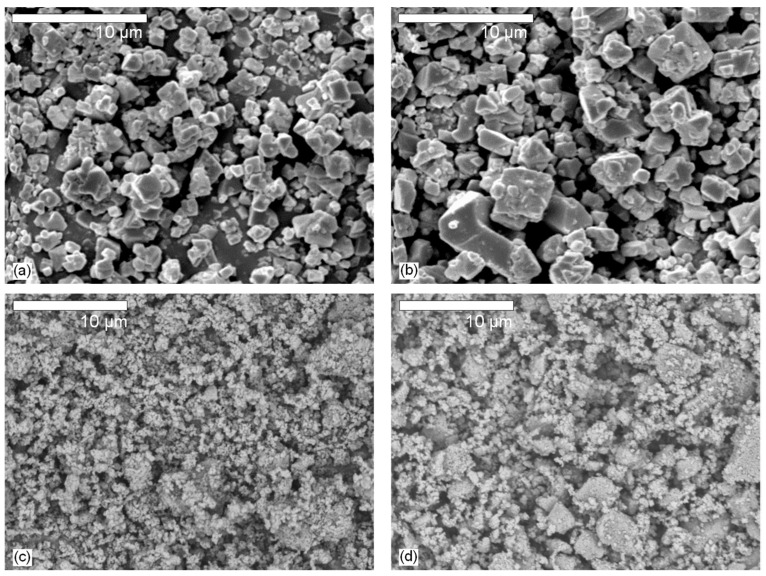
SEM images of aluminum lithium fluoride oxide (ALFO) (**a**,**b**) and LiAl_5_O_8_ (**c**,**d**). The preparation temperatures were 1200 °C for (**a**,**c**) and 1300 °C for (**b**,**d**).

Figure 3 shows the SEM images of 0.5% Cr^3+^-doped ALFO and LiAl_5_O_8_ prepared at 1200 °C and 1300 °C. The effect of treatment temperature did not appear to have a significant effect on the morphology; ALFO:Cr^3+^ was composed of angular grains with well-developed crystal faces. The grain size varied from sub-micrometers to several micrometers. On the other hand, the grains of LiAl_5_O_8_:Cr^3+^ did not have a definite shape and their size was several hundred nanometers, which formed agglomerates of a few micrometers in size. The difference in morphology between ALFO:Cr^3+^ and LiAl_5_O_8_:Cr^3+^ was attributed to the difference in the lithium sources. The melting points of LiF and Li_2_CO_3_ used to prepare ALFO and LiAl_5_O_8_ are 848 °C and 723 °C, respectively. The development of the crystal faces of the ALFO:Cr^3+^ grains strongly suggested that LiF formed the melt at elevated temperatures and acted as a self-flux to grow the crystalline grains. On the other hand, the indistinct shape of the grains observed in Figure 3c,d for LiAl_5_O_8_:Cr^3+^ indicated that no flux growth effect was expected, and thus Li_2_CO_3_ reacted with α-Al_2_O_3_:Cr^3+^ to form LiAl_5_O_8_:Cr^3+^ before forming the melt.

### 3.3. Photoluminescence Properties

**Figure 4:**
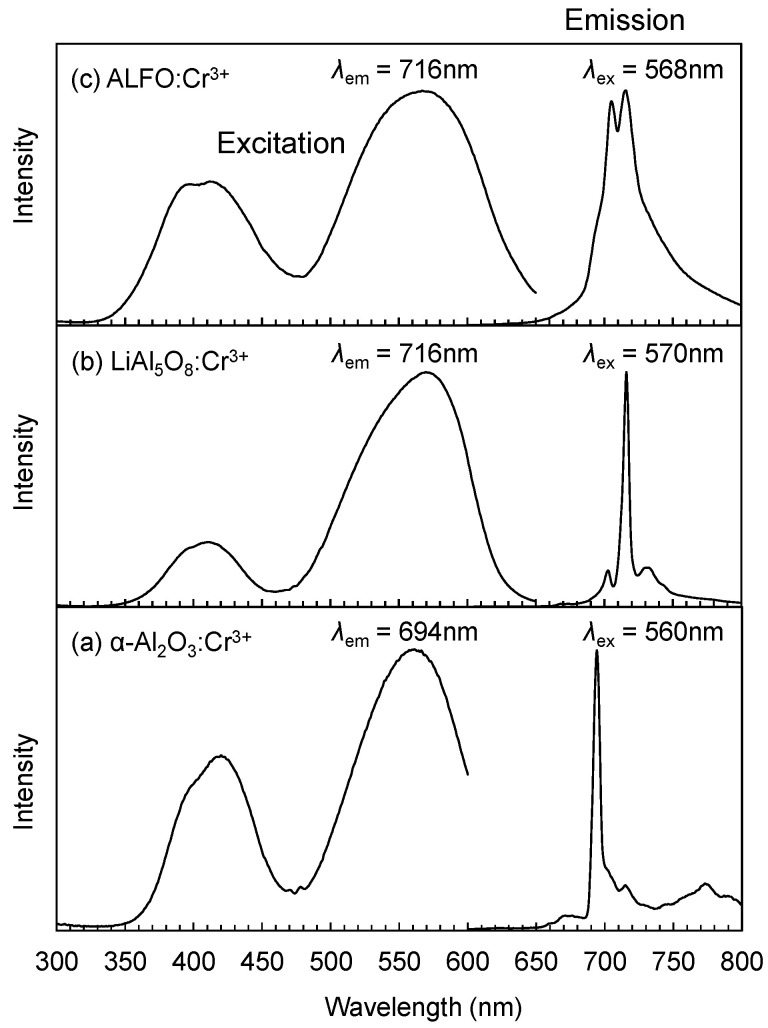
Emission and excitation spectra of 0.5% Cr^3+^ in α-Al_2_O_3_ (**a**), LiAl_5_O_8_ (**b**), and ALFO (**c**).

The emission and excitation spectra of 0.5% Cr^3+^ in ALFO, LiAl_5_O_8_, and α-Al_2_O_3_ prepared at 1200 °C are compared in Figure 4. The excitation spectra were similar to each other regardless of the type of the host crystal, and the excitation bands consisted of two broad peaks at around 420 nm and 570 nm. They corresponded to the transitions from the ground state ^4^*A*_2_ to the excitation states ^4^*T*_1_ (^4^*F*) and ^4^*T*_2_ (^4^*F*) in Cr^3+^ of the d^3^ state. The emission peaks depended on the host crystal. α-Al_2_O_3_:Cr^3+^ showed a typical line spectrum, named “R line”, at 694 nm with several small side peaks on the longer wavelength side, as found in the literature [9,44]; LiAl_5_O_8_:Cr^3+^ showed a sharp peak at 716 nm with smaller peaks at 703 nm and 730 nm which are also similar to those reported in the previous literature [19,45]. On the other hand, Cr^3+^ in ALFO characteristically exhibited a doublet peak at 708 and 716 nm, and the profile was broader than those of α-Al_2_O_3_ and LiAl_5_O_8_. Although the apparent difference between the crystal structures of ALFO and ordered LiAl_5_O_8_ shown by the Rietveld analysis was in the cation arrangement, the broad doublet peak in ALFO:Cr^3+^ indicated that the local environment around the luminescent center Cr^3+^ in ALFO was even more inhomogeneous than simply considering octahedral coordination by six neighboring oxygens.

**Figure 5:**
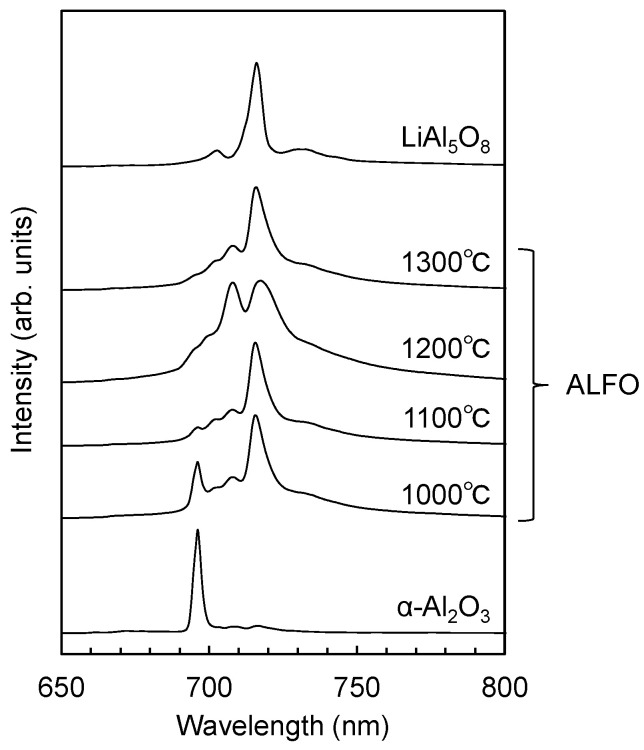
Variation of PL spectra of ALFO:Cr^3+^ with preparation temperature. The emission peaks of α-Al_2_O_3_:Cr^3+^ and LiAl_5_O_8_:Cr^3+^ prepared at 1200 °C are also shown for comparison.

Figure 5 shows the variation of the emission peaks of ALFO:Cr^3+^ with the treatment temperature and compares them with those of α-Al_2_O_3_:Cr^3+^ and LiAl_5_O_8_:Cr^3+^. The peak at 696 nm of the ALFO:Cr^3+^ sample prepared at 1000 °C was due to the unreacted Al_2_O_3_ residue (Figure S5 in Supplementary Materials), indicating that the coprecipitated Cr^3+^ was incorporated into the α-Al_2_O_3_ grains to form α-Al_2_O_3_:Cr^3+^. The peak at around 715 nm was specific to ALFO:Cr^3^ and was observed in ALFO:Cr^3+^ prepared at 1000–1300 °C. Interestingly, the rather broad doublet peak with peak tops at 708 nm and 716 nm was characteristically observed in the sample prepared at 1200 °C, and the detailed mechanism of the occurrence of the doublet peak is still unclear. It could be attributed to the difference in the local coordination environment around Cr^3+^ that did not appear in the average structure by XRD (Figure S5 in Supplementary Materials). Figure S5 in Supplementary Materials shows the XRD patterns of the ALFO:Cr^3+^ samples prepared at 1000–1300 °C. Residual unreacted α-Al_2_O_3_ was recognized in the sample prepared at 1000 °C, while the samples prepared at 1100–1300 °C were in the single phase.

**Figure 6:**
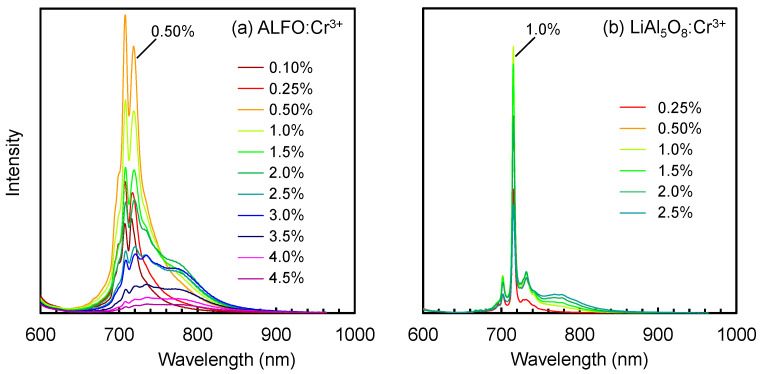
Emission peaks of ALFO:Cr^3+^ (**a**) and LiAl_5_O_8_:Cr^3+^ (**b**) as a function of Cr^3+^ concentration.

Figure 6 compares the PL spectra of ALFO:Cr^3+^ (a) and LiAl_5_O_8_:Cr^3+^ (b) at different Cr^3+^ concentrations. The emission intensity of ALFO:Cr^3+^ increased from Cr^3+^ = 0.1% to 0.5% and decreased above 0.5%. Above 2.5%, the intensity of the main doublet peak decreased while the shoulders developed around 780 nm. In the previous literature, it has been discussed that the broad emission of Cr^3+^ in the near-infrared region was due either to the ^4^*T*_2_ → ^4^*A*_2_ transition of Cr^3+^ placed in a weak crystal field, or to magnetic interactions between Cr^3+^ ions at the neighboring sites. According to the Tanabe–Sugano diagram [46,47,48], the first excited state in the *d*^3^ configuration of octahedral coordination is the ^4^*T*_2_ or ^2^*E* state, depending on the strength of the crystal field. The transition from the ^2^*E* state to the ^4^*A*_2_ ground state yields the line spectra typically observed for Cr^3+^ around 700 nm, while broad near-infrared emission is reported for the transition from the ^4^*T*_2_ state in the host materials with a weak crystal field [21,22,23,24,27,29,30]. Magnetic interactions also bring the red-shift of the emission [49], as exemplified by the so-called N-lines of Cr^3+^ emission [9,15] and the broad luminescence due to Cr^3+^-Cr^3+^ pairs observed in SrAl_11.88−*x*_Ga*_x_*O_19_:0.12Cr^3+^ by Rajendran et al., which was concluded from the decay time measurements [28]. As for the shoulder peak around 780 nm in ALFO:Cr^3+^, the origin was attributed to the magnetic interaction between Cr^3+^ ions from the fact that the change in the lattice parameter with Cr^3+^ addition, i.e., the change in the strength of the crystal field at the Cr^3+^ sites, was not very large (Figure S4 in Supplementary Materials), and that the interaction between Cr^3+^ ions was observed in the ESR signal at a high Cr^3+^ concentration as described in Section 3.4.

LiAl_5_O_8_:Cr^3+^ showed the maximum intensity at Cr^3+^ = 1.0%, and the change of the overall peak profile was less distinct than ALFO:Cr^3+^. The broad peak around 780 nm also developed in LiAl_5_O_8_:Cr^3+^ above 1.0%, but the intensity was relatively low.

**Figure 7:**
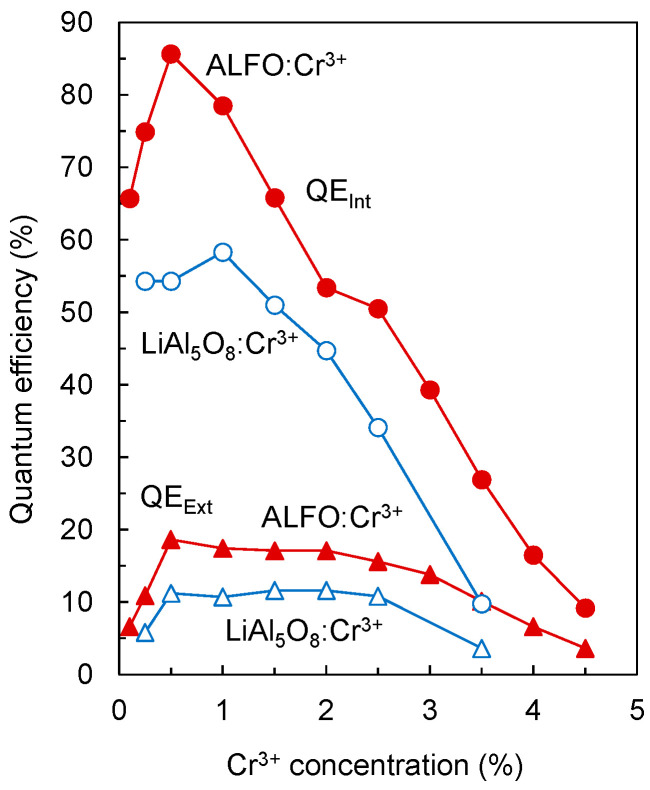
Internal and external quantum efficiencies of ALFO:Cr^3+^ and LiAl_5_O_8_:Cr^3+^ with different Cr^3+^ concentrations. QE_Int_ and QE_Ext_ represent internal and external quantum efficiencies, respectively.

Figure 7 shows the variation of the internal and external quantum efficiencies (QEs) of ALFO:Cr^3+^ and LiAl_5_O_8_:Cr^3+^ versus Cr^3+^ concentration. The circles and triangles indicate the internal and external quantum efficiencies QE_Int_ and QE_Ext_, respectively; the filled and unfilled marks indicate the QEs of ALFO:Cr^3+^ and LiAl_5_O_8_:Cr^3+^, respectively. ALFO:Cr^3+^ showed higher QEs than LiAl_5_O_8_:Cr^3+^. The QE_Int_ for ALFO was 85.6% at Cr^3+^ = 0.5%, higher than the maximum QE_Int_ of 58.3% for LiAl_5_O_8_ at Cr^3+^ = 1.0%. The highest QE_Ext_ was 18.6% for ALFO:Cr^3+^ and 11.6% for LiAl_5_O_8_:Cr^3+^. The enhancement of QEs of ALFO:Cr^3+^ was attributed to the high crystallinity suggested by the well-developed grains observed in SEM (Figure 3), the disruption of local symmetry around the luminescent center Cr^3+^ from the ideal octahedron with the introduction of F^−^ and vacancies on the O^2−^ sites, and the increased area of the PL peaks due to peak broadening.

### 3.4. Local Environments around Cr^3+^

Cr^3+^ prefers octahedral coordination in the host spinel lattice in both ALFO and LiAl_5_O_8_, whereas the different spectra suggested different local environments around Cr^3+^ in ALFO and LiAl_5_O_8_. Such aperiodic local structures are difficult to investigate using diffraction-based structural analysis, particularly for low-concentration dopants. In fact, the ALFO:Cr^3+^ samples prepared at the different temperatures showed substantially the same XRD patterns (Figure S5 in Supplementary Materials), but the emission peaks varied as shown in Figure 5.

**Figure 8:**
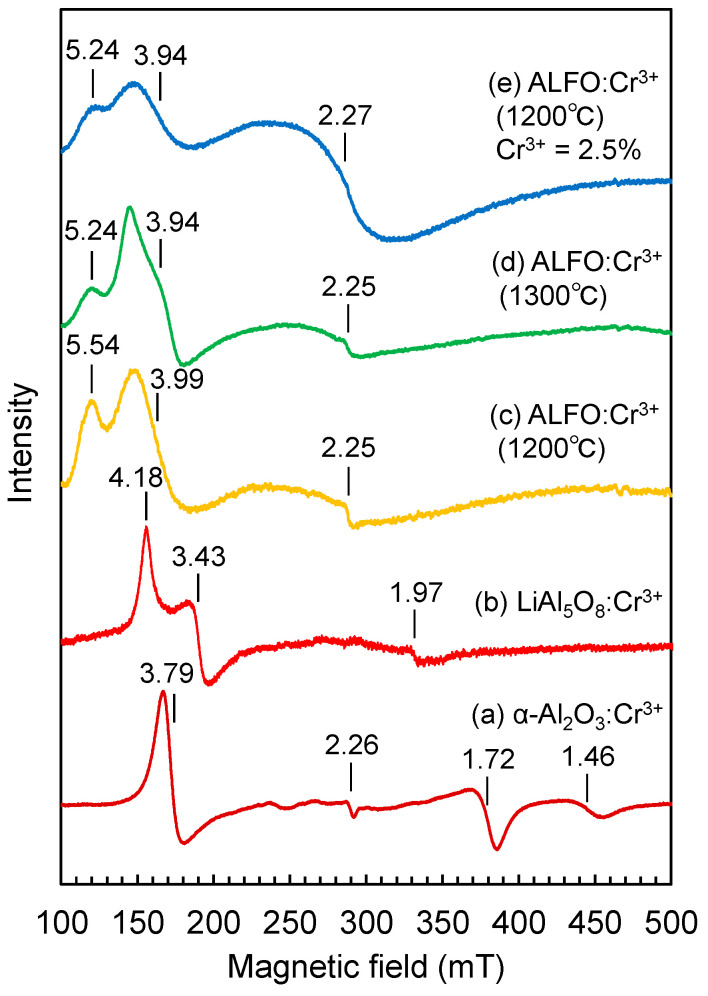
ESR signals of 0.5% Cr^3+^ in (a) α-Al_2_O_3_, (b) LiAl_5_O_8_, (c) ALFO prepared at 1200 °C, (d) ALFO prepared at 1300 °C, and (e) ALFO prepared at 1200 °C containing 2.5% Cr^3+^.

Cr^3+^ in the d^3^ configuration is an ESR active ion, and the ESR technique was expected to effectively detect the differences in the electronic structures affected by the coordination environment. Figure 8 compares the ESR spectra of 0.5% Cr^3+^ doped α-Al_2_O_3_ (a), LiAl_5_O_8_ (b), and ALFO (c) prepared at 1200 °C. Cr^3+^ in α-Al_2_O_3_ was focused on in the 1960s as a good model example for ESR measurements [44,50,51,52,53,54,55,56,57,58]. The ESR signal of Cr^3+^ in α-Al_2_O_3_ (Figure 8a) was consistent with those reported for Cr^3+^ in polycrystalline α-Al_2_O_3_ in the previous literature [50] and consisted of several peaks corresponding to *g* = 3.79, 2.26, 1.72, and 1.46. For octahedrally coordinated d^3^ ions placed in a magnetic field, the lowest energy state is the spin quartet, with the spin quantum number *s* = −3/2 along the direction of the magnetic field. The signal of a polycrystalline sample is the average of the spectra of individual crystallites, and the angular dependence of Cr^3+^ in single crystalline Al_2_O_3_ [59] indicated that the peaks at *g* = 3.79 and 2.26 were assigned to the *s* = −3/2 to −1/2 transition, and the peaks at *g* = 1.72 and 1.46 to the −1/2 to +1/2 transition and +1/2 to +3/2 transition, respectively (Appendix B) [50,51].

Cr^3+^ in LiAl_5_O_8_ showed essentially the same ESR signal (Figure 8b) as reported by Singh et al. [45] for LiAl_5_O_8_:Cr^3+^. Singh et al. assigned distinct peaks at *g* = 4.03 and 3.27 and smaller peaks at *g* = 5.44, 4.89, and 4.51 to the isolated Cr^3+^ ions and the resonance signal at *g* = 1.97 to the magnetic interaction between the Cr^3+^ ions, based on the literature [60,61].

The basic features of the ESR signal of Cr^3+^ in ALFO (Figure 8c) were similar to those of Cr^3+^ in LiAl_5_O_8_ (Figure 8b), but the profiles became broad and the signal positions shifted toward the low magnetic field side. The broadening of the ESR signal was attributed to an inhomogeneous local environment around the Cr^3+^ ions in ALFO. The CrO_6_ octahedra in ALFO are expected to be disturbed by the statistical distribution of Al^3+^ and Li^+^ on the adjacent cation sites. The shift of the ESR signal of ALFO toward the lower field side than LiAl_5_O_8_ indicated the increased zero-field splitting.

The PL spectra of Cr^3+^ in ALFO prepared at 1100 °C and 1300 °C were apparently similar to that of Cr^3+^ in LiAl_5_O_8_, but the ERS signal of Cr^3+^ in ALFO prepared at 1300 °C (Figure 8d) was different from that in LiAl_5_O_8_ prepared at 1200 °C (Figure 8b), indicating that the similarity in the PL spectra was not due to similar local environments around the luminescent center Cr^3+^ in these host materials.

Increasing the Cr^3+^ concentration to 2.5% resulted in broadening and enhancement of the ESR peak around *g* ~ 2.3 (Figure 8e), which was considered to reflect the magnetic interaction between Cr^3+^ ions, as Singh et al. discussed that *g* = 1.95 was due to exchange coupling of Cr^3+^-Cr^3+^ pairs [45]. The broadening of the peak at *g* ~ 1.96 with increasing Cr^3+^ concentration was also observed in Cr^3+^-containing phosphate glasses in the literature [62]. The presence of the magnetic interactions between Cr^3+^ ions at high concentrations was consistent with the discussion of the PL spectra, where the broad shoulder peak developed around 780 nm with increasing Cr^3+^ concentration (Figure 6a).

Since it was difficult to directly deduce the detailed local environment from the ESR signals, the local environment of isolated Cr^3+^ ions is discussed here using MD. The O-Cr-O bond angles of CrO_6_ octahedra in α-Al_2_O_3_ and ordered LiAl_5_O_8_ obtained in MD are tabulated in Table S2 in Supplementary Materials.

**Figure 9:**
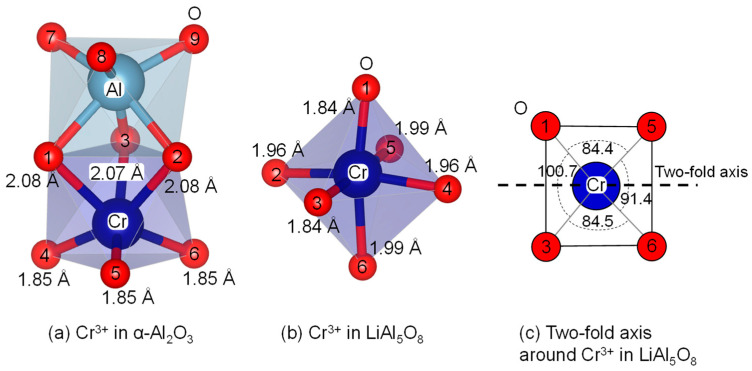
Local coordination environments reproduced by molecular dynamics simulations around Cr^3+^ in α-Al_2_O_3_ (**a**) and LiAl_5_O_8_ (**b**). (**c**) Two-fold axis is shown on the projected O1-O3-O6-O5 plane of the CrO_6_ octahedron in (**b**); note that the bond angles of O1-Cr-O5 (84.4°) and O3-Cr-O6 (84.5°) are not strictly coincident, reflecting thermal vibrations near the equilibrium position of each atom.

In α-Al_2_O_3_, Cr^3+^ was considered to be placed in a trigonal symmetry as suggested by McClure experimentally [63], which was also reproduced in our MD [39]. The previous literature referred to the rhombic distortion for CrO_6_ in LiAl_5_O_8_ with C_2_ symmetry [19,45]. Our MD result indicated that Cr^3+^ was arranged in a monoclinic symmetry that retained a single two-fold axis passing through the midpoints of O1 and O3, and of O5 and O6 (Figure 9b), and the two-fold axis is illustrated on the O1-O3-O6-O5 plane (c). Because of the complexity of the structure, including the disordered arrangement of cations and the presence of vacancies on the anion sites, MD for ALFO has not yet been performed; the local environment around Cr^3+^ in ALFO was inferred to be similar to that in LiAl_5_O_8_, but even more disordered.

## 4. Conclusions

Fluorine-doped lithium aluminate (ALFO) was prepared from LiF and Al_2_O_3_ at a ratio of 1:2. ALFO was a fluorine-doped form of LiAl_5_O_8_ with slightly lithium-rich compositions. The concentration of the incorporated fluorine was dependent on the preparation conditions; the chemical composition varied from Al_4.69_ Li_1.31_ F_0.28_ O_7.55_ prepared at 1100 °C to Al_4.83_ Li_1.17_ F_0.10_ O_7.78_ prepared at 1300 °C. ALFO and LiAl_5_O_8_ were isostructural with respect to having a spinel-type lattice and they were in a disorder–order relationship, where the regular arrangement of Li^+^ and Al^3+^ in ordered LiAl_5_O_8_ was disordered to a random arrangement with a slight preference of Li^+^ on the octahedral sites. The structural difference between ALFO and LiAl_5_O_8_ resulted in the different PL spectra for Cr^3+^. Cr^3+^ in ALFO showed deep red emission at around 700 nm, as did Cr^3+^ in LiAl_5_O_8_, but the peaks broadened. This was attributed to the disordered arrangement of Li^+^ and Al^3+^ on the adjacent cation sites, which led to ununiform local distortion of the CrO_6_ octahedra.

In conclusion, a new host material was proposed for deep red phosphors with the luminescent center Cr^3+^ excited by visible rays of blue (~410 nm) and green (~570 nm) light. This work revealed that the structural modification with a small amount of dopant (in this case, fluorine) led to variations in the luminescence properties and enhancement of PL even for the common lattice structure.

## Figures and Tables

**Table 1:** Chemical compositions of the fluorine-doped lithium aluminate (ALFO) host material prepared at different temperatures.

Preparation Temperature	Chemical Composition
1100 °C	Al_4.69_ Li_1.31_ F_0.28_ O_7.55_
1200 °C	Al_4.73_ Li_1.27_ F_0.17_ O_7.65_
1300 °C	Al_4.83_ Li_1.17_ F_0.10_ O_7.78_

## Data Availability

Data are available from the corresponding author upon reasonable request.

## Supplementary Materials

The following supporting information can be downloaded at: https://www.mdpi.com/article/10.3390/ma17020338/s1, Figure S1: XRD patters of aluminum lithium fluoride oxide (ALFO) samples prepared at 1100 °C, 1200 °C, and 1300 °C; Figure S2: The result of the Rietveld analysis for non-doped ALFO sample (Al_4.73_Li_1.27_F_0.17_O_7.65_) prepared at 1200 °C; Figure S3: Perspective view of the crystal structures of ordered LiAl_5_O_8_ (a) and aluminum lithium fluoride oxide (ALFO) (b) in the range *x* = 0.2 to 0.6 along <100>, illustrating the difference in the arrangement of the tetrahedra and the octahedra in these host materials; Figure S4: XRD patterns of aluminum lithium fluoride oxide (ALFO) samples prepared at 1200 °C with different Cr^3+^ concentrations; Figure S5: XRD patterns of aluminum lithium fluoride oxide (ALFO) samples prepared at different temperatures: Figure S6: Comparison of XRD patterns of disordered spinel (ALFO), ordered spinel (LiAl_5_O_8_), and partially ordered LiAl_5_O_8_ prepared by quenching from 1400 °C; Table S1: The structural parameters refined in the Rietveld analysis for non-doped ALFO sample (Al_4.73_Li_1.27_F_0.17_O_7.65_) prepared at 1200 °C; Table S2: Bond angles in CrO_6_ octahedra in α-Al_2_O_3_ and ordered LiAl_5_O_8_ after relaxation by MD; Table S3: Assignment of the ESR peaks of the polycrystalline sample of Cr^3+^ in α-Al_2_O_3_.

## Appendix A

According to the previous literature [19,40,41,42], LiAl_5_O_8_ underwent the phase transition between the low-temperature ordered (II) and high-temperature disordered (I) forms at 1295 °C. However, in our experiments, quenching of LiAl_5_O_8_ samples prepared from Li_2_CO_3_ and α-Al_2_O_3_ into liquid nitrogen from 1400 °C did not result in the fully disordered phase of LiAl_5_O_8_. The quenching resulted in partial Al^3+^ and Li^+^ disorder and reduced the peak intensity originating from the cation ordering, indicated by ▼ in Figure 1 and Figure S6 in Supplementary Materials. Figure S6 in Supplementary Materials compares the XRD patterns of ordered LiAl_5_O_8_ prepared at 1400 °C, partially ordered LiAl_5_O_8_ quenched from 1400 °C, and ALFO. The reason that disordered LiAl_5_O_8_ was not obtained in this work was that the samples were not started from the nitrate gel precursor [19,40,41,42], and/or a highly sensitive semiconductor X-ray detector, which is supposed to achieve diffraction intensities 100 times higher than those of conventional scintillation counters [64], was used.

ALFO is similar to disordered LiAl_5_O_8_ with respect to the random distribution of Li^+^ and Al^3+^ on the tetrahedral and octahedral cation sites; the incorporation of fluorine from the starting material LiF specifically leads to disorder in the Al and Li arrangement in ALFO.

## Appendix B

According to de Biasi [59], the ESR peaks of a polycrystalline α-Al_2_O_3_:Cr^3+^ sample occur at *θ* ≈ 40° and 90°, the angle (*θ*) between the direction of the applied magnetic field and the *z* axis. From the angle dependence of the ESR signal of a Cr^3+^-doped α-Al_2_O_3_ single crystal [51], the peaks were assigned as shown in Table S3 in Supplementary Materials.
