# Supplementary material for: Deep Red Photoluminescence from Cr3+ in Fluorine-Doped Lithium Aluminate Host Material
Source: Materials (Basel). 2024 Jan 10;17(2):338. doi: 10.3390/ma17020338 (PMC10817566; doi:10.3390/ma17020338)
Supplement: Supplementary file 1 [file materials-17-00338-s001.zip › materials-2777862-supplementary.pdf]

materials

Supplementary Materials

Deep Red Photoluminescence from Cr^{3+} in Fluorine-Doped Lithium Aluminate Host Material

Yuki Kamada [1], Ryusei Hayasaka [1], Kento Uchida [1], Taisei Suzuki [1], Takahiro Takei [2], Mamoru Kitaura [3], Hiroko Kominami [4], Kazuhiko Hara [5] and Yuta Matsushima [1,*]

[1] Applied Chemistry, Chemical Engineering, and Biochemical Engineering, Yamagata University, Yonezawa 992-8510, Japan
[2] Center for Crystal Science and Technology, University of Yamanashi, Kofu 400-0021, Japan
[3] Faculty of Science, Yamagata University, Yamagata 990-8560, Japan
[4] Faculty of Engineering, Shizuoka University, Hamamatsu 432-8561, Japan
[5] Research Institute of Electronics, Shizuoka University, Hamamatsu 432-8011, Japan
* Correspondence: ymatsush@yz.yamagata-u.ac.jp; Tel.: +81-238-26-3165

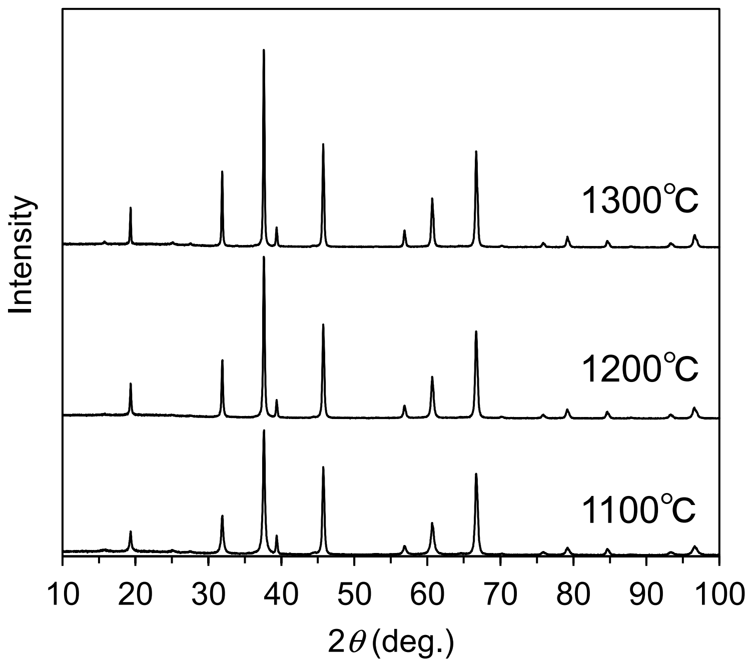

Figure S1 XRD patters of aluminum lithium fluoride oxide (ALFO) samples prepared at 1100°C, 1200°C, and 1300°C.

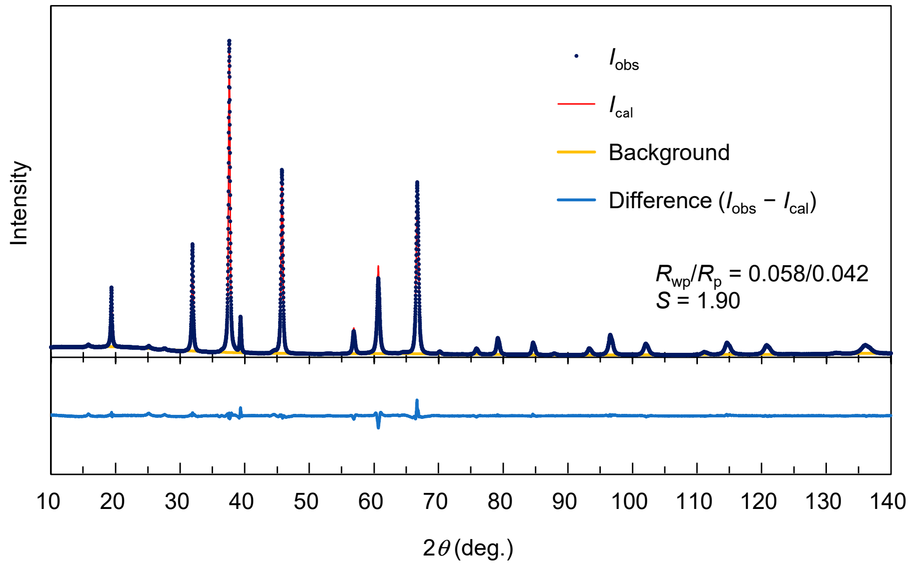

Figure S2 The result of the Rietveld analysis for non-doped ALFO sample ($Al_{4.73}Li_{1.27}F_{0.17}O_{7.65}$) prepared at 1200 °C.

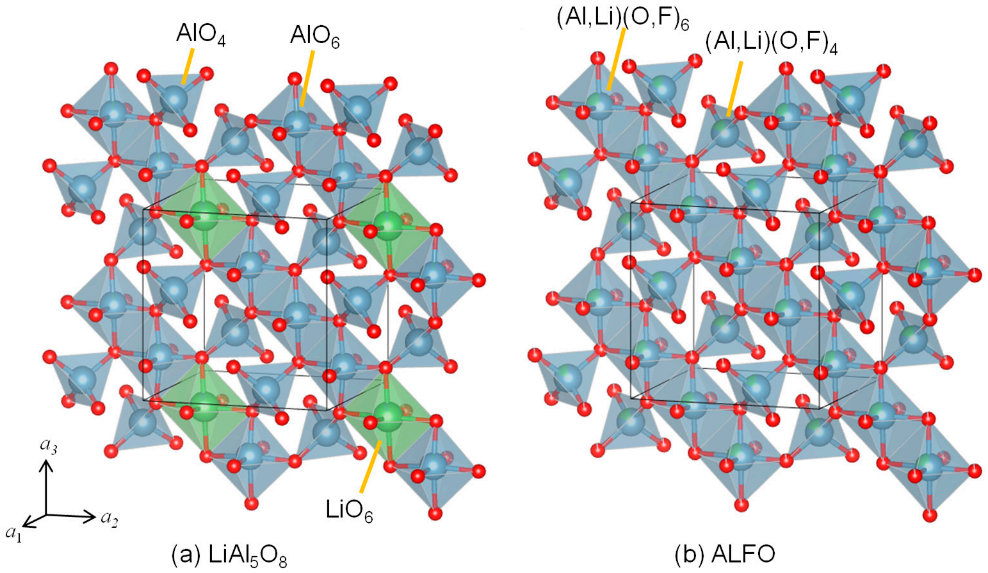

Figure S3 Perspective view of the crystal structures of ordered $LiAl_5O_8$ (a) and aluminum lithium fluoride oxide (ALFO) (b) in the range x = 0.2 to 0.6 along <100>, illustrating the difference in the arrangement of the tetrahedra and the octahedra in these host materials.

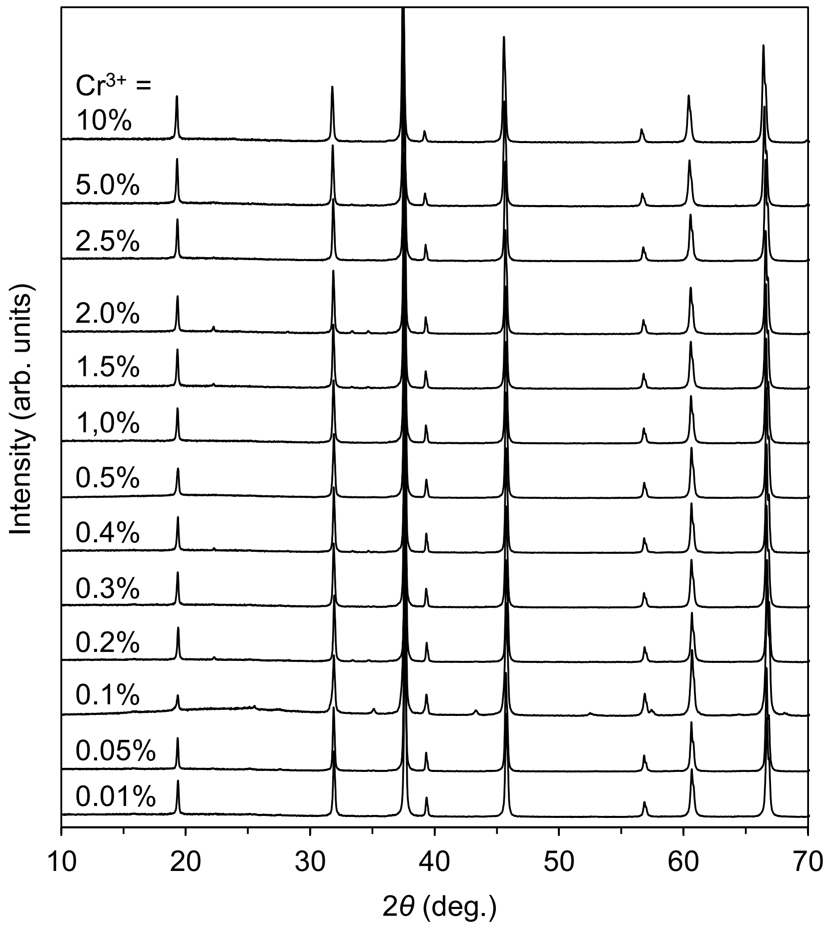

Figure S4 XRD patterns of aluminum lithium fluoride oxide (ALFO) samples prepared at 1200°C with different Cr^{3+} concentrations.

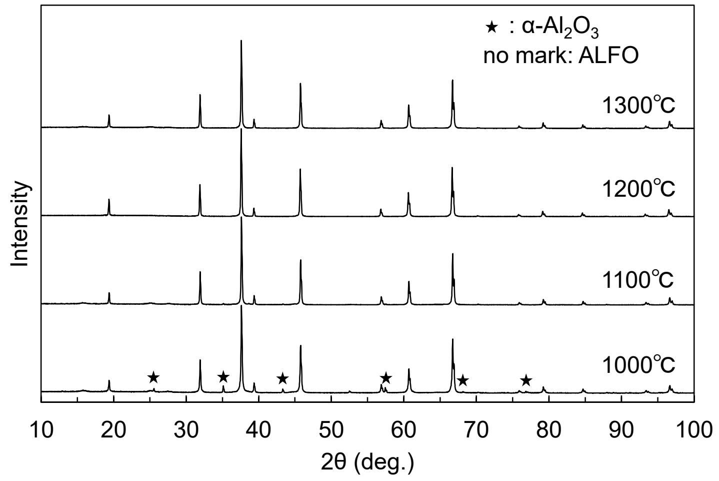

Figure S5 XRD patterns of aluminum lithium fluoride oxide (ALFO) samples prepared at different temperatures.

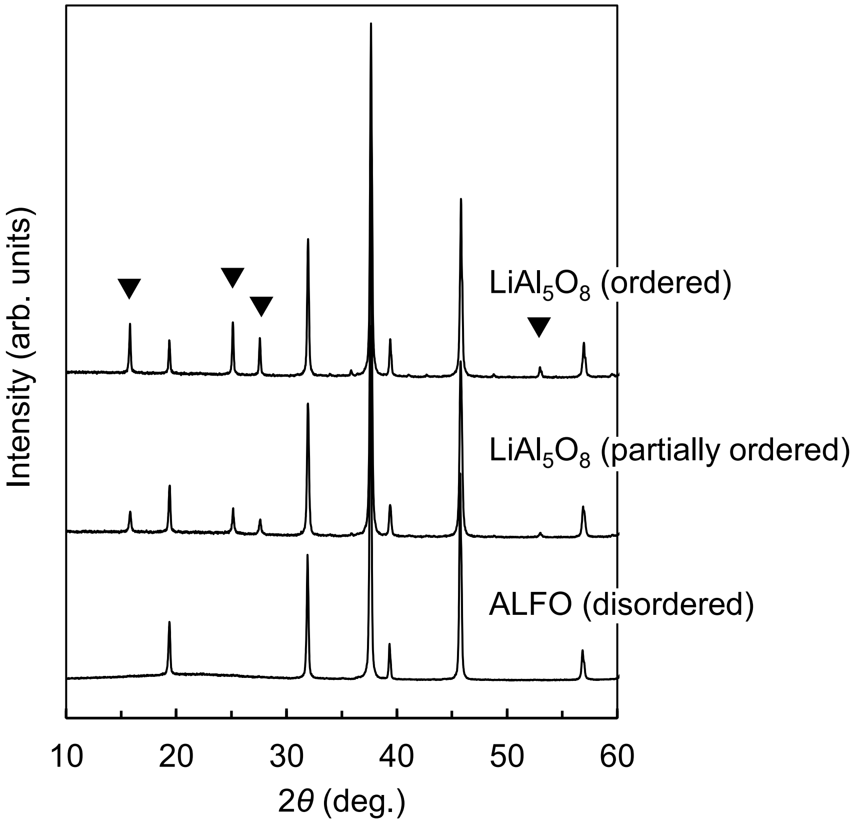

Figure S6 Comparison of XRD patterns of disordered spinel (ALFO), ordered spinel ($LiAl_5O_8$), and partially ordered $LiAl_5O_8$ prepared by quenching from 1400°C.

Table S1 The structural parameters refined in the Rietveld analysis for non-doped ALFO sample ($Al_{4.73}Li_{1.27}F_{0.17}O_{7.65}$) prepared at 1200°C.

**Space group**		*Fd–3m*				
**Lattice constant**		7.9222(8) Å				
R_{wp} / R_p / S		0.058 / 0.042 / 1.90				
**Atom**	**Wycoff position**	**Occupancy, *g***	***x***	***y***	***z***	***B* (Å²)**
Al1	8a	0.817(1)	0	0	0	0.67(1)
Li1	8a	0.183<br>= 1 – *g*(Al1)	0	0	0	= *B*(Al1)
Al2	16d	0.774	5/8	5/8	5/8	0.79(1)
Li2	16d	0.226<br>= 1 – *g*(Al2)	5/8	5/8	5/8	= *B*(Al2)
O	32e	0.9563	0.38139(4)	= *x*	= *x*	0.72(1)
F	32e	0.0213	= *x*(O)	= *x*(O)	= *x*(O)	= *B*(O)

Table S2 Bond angles in CrO_6 octahedra in α-Al_2O_3 and ordered $LiAl_5O_8$ after relaxation by MD.

**α-Al_2O_3:Cr^{3+}**		**$LiAl_5O_8$:Cr^{3+}**	
**Bond angles (°)**		**Bond angle (°)**	
76.3	O1-Cr-O2, O2-Cr-O3, O2-Cr-O3	81.3	O2-Cr-O5, O4-Cr-O6
84.8-9	O1-Cr-O4, O2-Cr-O5, O3-Cr-O6	84.4	O1-Cr-O5, O3-Cr-O6
89.5-7	O1-Cr-O5, O2-Cr-O6, O3-Cr-O4	86.5-6	O2-Cr-O6, O4-Cr-O5
105.2-3	O4-Cr-O5, O4-Cr-O6, O5-Cr-O6	90.9-91.4	O1-Cr-O4, O2-Cr-O3, O5-Cr-O6
		100.2-7	O1-Cr-O2, O1-Cr-O3, O3-Cr-O4

Table S3 Assignment of the ESR peaks of the polycrystalline sample of Cr^{3+} in α-Al_2O_3.

**α-Al_2O_3:Cr^{3+}**		
***g* value**	**Transition**	**Angle (*θ*)**
3.79	−3/2 → −1/2	90°
2.26	−3/2 → −1/2	40°
1.72	−1/2 → +1/2	40°
1.46	+1/2 → +3/2	90°
